# Distinguishing Axillary Lymphadenopathy after COVID-19 Vaccination from Malignant Lymphadenopathy

**DOI:** 10.3390/jcm13123387

**Published:** 2024-06-09

**Authors:** Shintaro Yamanaka, Keiko Tanaka, Masao Miyagawa, Teruhito Kido, Shinji Hasebe, Shoichiro Yamamoto, Tomomi Fujii, Kazuto Takeuchi, Yoshihiro Yakushijin

**Affiliations:** 1 Department of Clinical Oncology, Ehime University Graduate School of Medicine, Toon 791-0295, Japan; aidaisintaro515@gmail.com (S.Y.); hasebe.shinji.yw@ehime-u.ac.jp (S.H.); shoichiroy@gmail.com (S.Y.); tfujii@m.ehime-u.ac.jp (T.F.); 2 Department of Epidemiology and Public Health, Ehime University Graduate School of Medicine, Toon 791-0295, Japan; tanaka.keiko.jn@ehime-u.ac.jp; 3 Department of Radiology, Ehime University Graduate School of Medicine, Toon 791-0295, Japan; miyagawa848@gmail.com (M.M.); kido.teruhito.mu@ehime-u.ac.jp (T.K.); 4 Department of Clinical Laboratory, Ehime Prefectural University of Health Sciences, Tobe 791-2101, Japan; kazutake0415@gmail.com

**Keywords:** COVID-19, vaccine, axillary-lymphadenopathy, ^18^F-FDG PET

## Abstract

**Objectives**: To study the differences between malignant hypermetabolic axillary lymphadenopathy (MHL) and COVID-19 vaccine-associated axillary hypermetabolic lymphadenopathy (VAHL) using clinical imaging. **Methods**: A total of 1096 patients underwent Positron Emission Tomography-Computed Tomography (PET-CT) between 1 June 2021 and 30 April 2022 at Ehime University Hospital. In total, 188 patients with axillary lymphadenopathy after the COVID-19 vaccination were evaluated. The patients were classified into three groups such as VAHL (*n* = 27), MHL (*n* = 21), and equivocal hypermetabolic axillary lymphadenopathy (EqHL; *n* = 140). Differences in lymph node (LN) swellings were statistically analyzed using clinical imaging (echography, CT, and ^18^F-FDG PET). **Results**: MHL included a higher female population (90.5%) owing to a higher frequency of breast cancer (80.9%). Axillary LNs of MHL did not show any LN fatty hilums (0%); however, those of VAHL and EqHL did (15.8 and 36%, respectively). After the logistic regression analysis of the patients who had axillary lymphadenopathy without any LN fatty hilums, the minor axis length and ellipticity (minor axis/major axis) in the largest axillary LN, SUVmax, and Tissue-to-Background Ratio (TBR) were useful in distinguishing malignant lymphadenopathies. A receiver-operating characteristic (ROC) analysis indicated that a cut-off value of ≥7.3 mm for the axillary LN minor axis (sensitivity: 0.714, specificity: 0.684) and of ≥0.671 for ellipticity (0.667 and 0.773, respectively) in the largest LN with the highest SUVmax and TBR were predictive of MHL. **Conclusions**: Axillary lymphadenopathy of the minor axis and ellipticity in LN without fatty hilums may be useful to be suspicious for malignancy, even in patients who have received COVID-19 vaccination. Further examinations, such as ^18^F-FDG PET, are recommended for such patients.

## 1. Introduction

Since the commencement of the COVID-19 vaccination campaign in Japan in February 2021, over 38 million doses of COVID-19 vaccination have been administered, and over 68% of the Japanese population has already been vaccinated three or more times [1]. Among the various side effects, post-vaccination axillary lymphadenopathy, specifically on the side of the inoculation, has been reported as a clinical problem [2,3,4,5]. As with other mRNA vaccines, antigen migration induced by vaccination from the injection site to draining nodes can result in lymph node swelling. Recent clinical observations and reports in patients after a COVID-19 vaccination have indicated that the frequency of axillary lymphadenopathy ranges from 0.3% to 16% post-vaccination and the incidence generally increases with the number of subsequent vaccinations [6]. Detection of lymphadenopathy on various imaging modalities, including ultrasonography (US), CT, MRI, and 18F-FDG PET, has been documented in several case reports and small cohort studies, and the incidence [7,8,9,10,11,12,13,14] and the immunological mechanisms behind its development [15,16,17] are currently being clarified. For patients with cancer or a history of cancer, distinguishing between reactive and malignant lymphadenopathy is a critical issue in clinical practice. Several recent studies have attempted to distinguish the pathological and clinical findings of lymphadenopathy between COVID-19 vaccination and other diseases and to evaluate the significance of early diagnosis among the entities [18,19,20,21]. To date, most of the studies have focused their attention on metabolically active axillary lymphadenopathy frequency and dimension, whilst studies on anatomical criteria, trying to characterize them on imaging modalities, are scarce. The differentiation of the imaging characteristics between COVID-19-associated (reactive) lymphadenopathy and malignancy is not entirely understood. The aim of the present study was to discern the differences between reactive (vaccine-associated hypermetabolic lymphadenopathy [VAHL]) and malignant (malignant hypermetabolic lymphadenopathy [MHL]) axillary lymphadenopathy based on the analysis of clinical imaging data.

## 2. Materials and Methods

### 2.1. Study Design and Patients

In this retrospective single-center study, patients with proven malignant tumors who underwent echographic examination and CT scan and/or an 18F-FDG PET-CT imaging at the Nuclear Medicine Department of Ehime University Hospital were eligible for inclusion in the study. Patients who were suspected and confirmed to have reactive unilateral axillary lymphadenopathy at the initial presentation after COVID-19 vaccination and who received clinical follow-up and imaging examinations were also eligible for inclusion.

### 2.2. Criteria for Unilateral Lymphadenopathy and Data Collection

**Figure 1:**
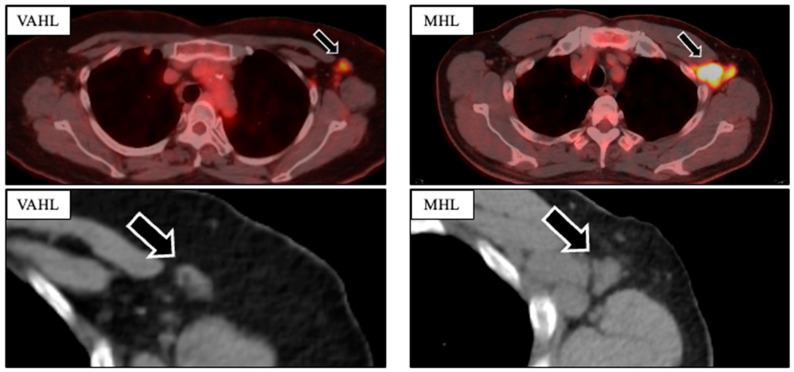
Comparison of PET-CT imaging findings in VAHL and MHL to assess the morphology (minor axis, major axis, presence of fatty hilum) and ^18^F-FDG uptake intensity of the axillary lymph nodes.

Data from patients aged ≥ 20 years who underwent Positron Emission Tomography-Computed Tomography (PET-CT) between 1 June 2021 and 30 April 2022 were collected from the electronic medical records of Ehime University Hospital. To assist in the clinical interpretation of PET-CT scans, the hospital’s Department of Nuclear Medicine started routinely documenting details of COVID-19 vaccination (date of vaccination and injection site) in June 2021. These data were used in the present study. During the study period, a total of 1096 patients underwent a PET-CT, of whom 201 were diagnosed with unilateral lymphadenopathy. Uptake intensity in lymph nodes was measured as the maximum standardized uptake value (SUVmax) on the PET-CT images. The SUVmax value was rounded up to the first decimal place [22]. Lymph node size (mm) was measured on CT images as cortical thickness, minor axis diameter, and major axis diameter. A diagnosis of significant axillary lymph node swelling or notable FDG accumulation in the axillary lymph node (ALN) was reached after confirmation by two or more specialists in nuclear medicine (Figure 1).

**Figure 2:**
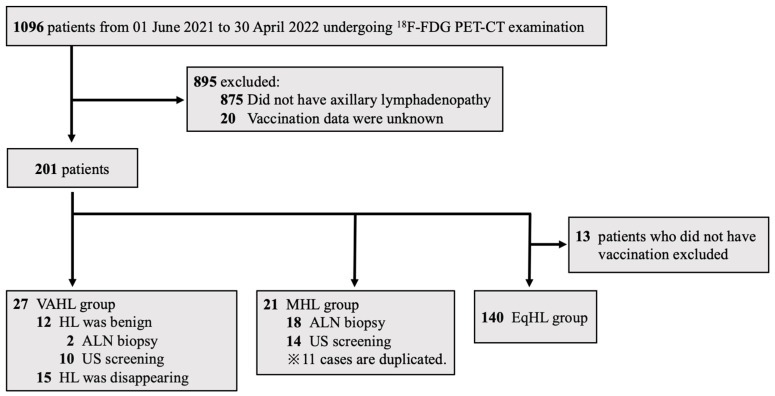
Grouping of the three categories: VAHL, MHL, and EqHL. ^18^F-FDG PET-CT = 18F-fluorodeoxyglucose positron emission tomography-computed tomography; HL = hypermetabolic lymphadenopathy; ALN = axillary lymph node; VAHL = vaccine-associated hypermetabolic lymphadenopathy; MHL = malignant hypermetabolic lymphadenopathy; EqHL = equivocal hypermetabolic lymphadenopathy.

Patients were classified into three ALN groups based on careful clinical observation: benign lymph nodes associated with COVID-19 vaccination (vaccine-associated hypermetabolic lymphadenopathy, VAHL group), lymph node metastasis from a malignant tumor (malignant hypermetabolic lymphadenopathy, MHL group) after or not after COVID-19 vaccination, and equivocal hypermetabolic lymphadenopathy (EqHL group) after vaccination. In the VAHL group, we examined the ipsilateral lymph nodes after COVID-19 vaccination. The VAHL group included patients who developed lymphadenopathy within 10 weeks following vaccination and met at least one of the following criteria: lymph nodes assessed as benign according to echography and/or biopsy or documentation of decreased nodal size over time. These criteria were based on the findings of a previous study that demonstrated a significant reduction in unilateral axillary lymphadenopathy at least 10 weeks post-vaccination [10]. The MHL group consisted of patients with a confirmed vaccination status from medical records and a diagnosis of malignant lymphadenopathy based on the findings of an echographic examination or lymph node biopsy. The EqHL group included vaccinated patients whose axillary lymphadenopathy could not be conclusively classified as either MHL or VAHL (Figure 2).

Patients’ sex, age, date and site of vaccination, primary disease, purpose of the PET-CT imaging, and blood glucose levels at the time of imaging were collected from the electronic records and analyzed.

### 2.3. Echographic Examination

Axillary US was performed using an Aplio a550 CUS-AA550 (Canon Medical Systems Inc., Ohtawara-shi, Japan) unit with a 12 MHz linear transducer (18L7; PLT-1204BT, Canon Medical Systems Inc.). Detailed information on the axillary lymph nodes was obtained on B-mode and color Doppler US images. A lymph node abnormality was defined as focal bulging of the cortex > 3 mm or effacement of the hilum (partial [<100%] or complete [100%]) on ultrasound. Suspected lymph node (LN) metastasis was defined based on the following criteria: nodal roundness, absence of fatty tissue in the hilum and the node, diffuse or localized cortical thickening > 3 mm, and peripheral blood flow signals within the lymph nodes. The echographic procedures were performed by surgical oncologists with more than 3 years of experience in breast surgery.

### 2.4. ^18^F-FDG PET-CT Imaging

^18^F-FDG PET-CT imaging was performed using an integrated digital PET-CT scanner (Discovery MI; GE Healthcare, Milwaukee, WI, USA) in all patients. Patients were fasted for at least 6 h and had a blood glucose level of 80–120 mg/dL before intravenous administration of ^18^F-FDG (3.7 MBq/kg). The total examination time for the PET-CT was approximately 20 min. All PET images were reconstructed using a three-dimensional time-of-flight weighted line-of-response row-action maximum-likelihood algorithm, with attenuation correction using a CT attenuation map. Integrated PET and CT images were reviewed on an Advantage Workstation Server 3.2 Ext. 3.2 (GE Healthcare). The display field of view was 60 × 60 cm, consisting of 256 × 256 matrixes. Voxel size was 2.34 × 2.34 × 2.79 mm.

### 2.5. PET-CT Imaging Analysis

**Figure 3:**
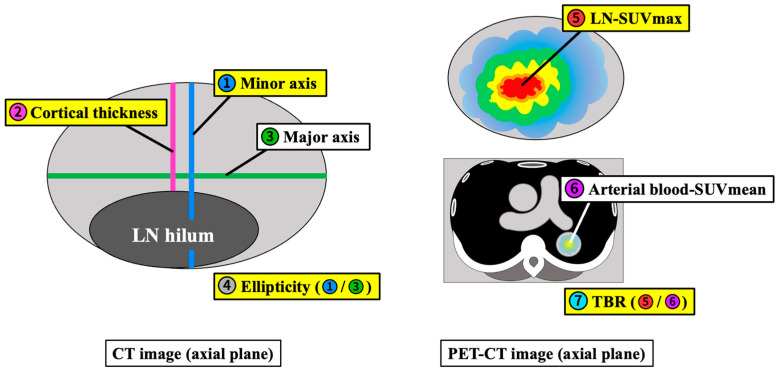
Schemas of the measurement methods. Cortical thickness; length of the minor axis minus the lymphatic hilum, ellipticity; minor axis length divided by major axis length, LN-SUVmax; SUVmax of hypermetabolic axillary lymphadenopathy, arterial blood-SUVmean; SUVmean of arterial blood in the thoracic aorta at the level of the axilla (B-SUVmean), TBR; LN-SUVmax divided by B-SUVmean. All length and thickness measurements are in millimeters.

The PET and CT images were evaluated visually and quantitatively. For each PET-CT dataset, the morphological features and ^18^F-FDG uptake of hot ALNs were carefully identified, and a volumetric region of interest (VOI) was drawn on each detectable ALN. The following measurements were obtained: length of the minor axis minus the lymphatic hilum (cortical thickness), minor axis length divided by major axis length (ellipticity), SUVmax of hypermetabolic axillary lymphadenopathy (LN-SUVmax), SUVmean of arterial blood in the thoracic aorta at the level of the axilla (B-SUVmean), and the ratio of tumor uptake to background calculated by dividing the LN-SUVmax by the B-SUVmean (Tissue-to-Background Ratio: TBR) (Figure 3).

### 2.6. Statistical Analysis

Normally distributed continuous variables were presented as the mean ± standard deviation (SD). Categorical variables are presented as numbers and percentages. The Student’s *t*-test was used for normally distributed continuous variables. A one-way analysis of variance (ANOVA) was used to compare the three groups. A chi-squared test was used for categorical variables. A multivariate model of the logistic regression model was used to evaluate the differentiating factors between vaccine-associated and malignant lymphadenopathy. The quantification of the effect size of the differentiating factors was performed using the ratio (OR) with a 95% confidence interval (95%CI). Statistical significance was defined as *p* < 0.05. A receiver-operating characteristic (ROC) curve analysis was performed to determine the cut-off value with the best combination of sensitivity and specificity. Statistical analysis was performed using the SAS software package version 9.4 (SAS Institute Inc., Cary, NC, USA).

## 3. Results

### 3.1. Patient Demographics

Table 1 presents the patient demographics according to the group. The mean age was 63.9 (±14.6 SD) years, with no significant difference noted across the three groups (*p* = 0.42). The proportion of females was the highest in the MHL group (90.5%), probably because 80.9% of the patients in this group had breast cancer. No significant differences were observed in age, disease distribution, or timing of imaging tests between the VAHL and EqHL groups (*p* > 0.05). More than half of the patients in all three groups underwent a PET-CT examination before treatment. The vaccination rate in the MHL group was 76.2%.

### 3.2. Qualitative Imaging Analysis (Ultrasonography and/or CT)

Table 2 shows the prevalence of a fatty hilum within the LNs of interest in all the groups. A fatty hilum was detected in 23.4% (44/188) of the LNs in all patients, including 30% (8/27) in the VAHL group, 0% (0/21) in the MHL group, and 25.7% (36/140) in the EqHL group. Chi-square testing indicated significant differences between the VAHL and MHL groups and between the MHL and EqHL groups (*p* < 0.01). This finding suggests that the presence or absence of a fatty hilum is an important factor in the differential diagnosis of benign and malignant axillary lymphadenopathies.

### 3.3. Quantitative LN Analysis on CT

**Figure 4:**
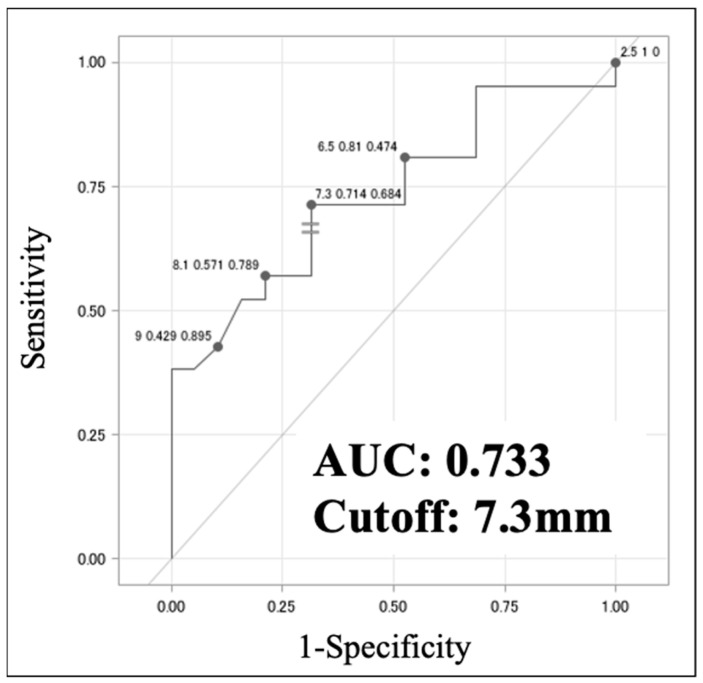
Receiver-operating characteristic (ROC) analysis of minor axis length between the VAHL and MHL groups. AUC indicates the area under the ROC curve.

In evaluating lymph nodes on CT images, the node with the longest minor axis was targeted. A comparison of the VAHL and MHL groups by univariate analysis revealed a statistically significant difference in minor axis length, which was also identified as an independent predictor of MHL by multivariate analysis (Table 3a). Although univariate analysis revealed significant differences in minor axis length and ellipticity between the MHL and EqHL groups, only the minor axis length was an independent predictor in the multivariate analysis (Table 3b). The ROC analysis identified an optimal cut-off for a minor axis length of 7.3 mm (sensitivity 0.714, specificity 0.684, AUC 0.733) for discriminating VAHL from MHL (Figure 4). These results suggest that an axillary lymph node length of >7.3 mm is a potential sign of malignancy.

### 3.4. Quantitative LN Analysis on PET-CT

**Figure 5:**
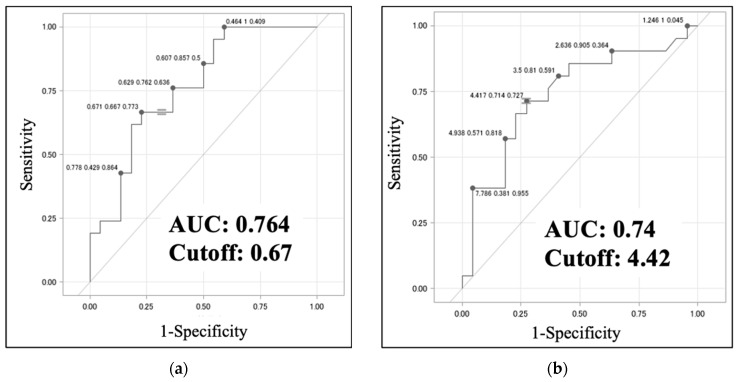
Receiver-operating characteristic (ROC) analysis of (**a**) ellipticity and (**b**) TBR between the VAHL and MHL groups. AUC indicates the area under the ROC curve.

For the assessment of lymph nodes on ^18^F-FDG PET-CT images, the node with the highest SUVmax was selected and analyzed. The TBR was measured in addition to the minor axis length and ellipticity. A comparison of the VAHL and the MHL groups using univariate analysis revealed statistically significant differences in minor axis length, ellipticity, and TBR (*p* < 0.01). In the multivariate analysis adjusted for age and blood glucose level at the time of PET-CT examination, ellipticity was identified as an independent predictor of MHL (*p* < 0.05) (Table 4a). A comparison of the MHL and EqHL groups by univariate analysis also identified statistically significant differences in the minor axis length, ellipticity, and TBR (*p* < 0.01), and the TBR showed a significant difference between the MHL and EqHL groups by multivariate analysis (*p* < 0.05) (Table 4b). The ROC analysis identified optimal cut-off values of 0.67 (sensitivity, 0.667; specificity, 0.773; AUC, 0.764) for LN ellipticity and 4.42 (sensitivity, 0.714; specificity, 0.727; AUC, 0.74) for the TBR of LNs for distinguishing between the VAHL and MHL groups (Figure 5a,b). These results indicate that in ALN swelling, ellipticity values > 0.67 and TBR values > 4.42 are possible signs of malignancy.

## 4. Discussion

The positive protective effect of vaccines is sometimes accompanied by unintended adverse effects, most of which are transient and minor. Lymph node enlargement following vaccination is related to locally activated antigens that accumulate at the injection site and later migrate to the draining nodes. The COVID-19 vaccination is administered intramuscularly into the deltoid muscle; vaccination-associated adenopathy typically occurs in the axilla and supraclavicular region. For patients with cancer or a history of cancer, distinguishing between reactive and malignant lymphadenopathy is a critical issue in clinical practice. The present study evaluated whether unilateral axillary VAHL after the COVID-19 vaccination can be differentiated from MHL in a clinical setting. We identified patients who exhibited unilateral axillary lymphadenopathy, confirmed their diagnoses based on complete clinical follow-up, and performed qualitative and quantitative assessments of the lymph nodes using the imaging techniques of ultrasonography, CT, and PET-CT. We then investigated useful factors for differentiating VAHL from MHL and evaluated whether imaging tests alone could facilitate diagnosis.

We first investigated whether the presence of a fatty hilum in the lymph nodes could serve as a useful finding for distinguishing between VAHL and MHL, utilizing non-invasive or minimally invasive imaging techniques (ultrasonography and non-contrast CT). We found that no patient with a fatty hilum presented with MHL (Table 2), suggesting that the presence of a fatty hilum could be a determinant of benign lymphadenopathy. In metastatic lymphadenopathy, the absence of a fatty hilum indicates the disruption of the normal lymph node structures. Previous studies have also reported the utility of the presence of a fatty hilum in differentiating between benign and malignant lymphadenopathy [23,24].

Second, in cases in which the fatty hilum is not detected in a lymph node, objective and quantitative evaluation using non-contrast CT might be necessary for diagnosis. Our analysis indicates that minor axis length is the next most important factor in reaching a diagnosis. The present analysis found that if the minor axis length of the largest enlarged lymph node was ≥7.3 mm on CT, it was significantly more likely to be MHL than VAHL (Figure 4). We consider that this cut-off value is more clinically useful compared to size classified as pathologically enlarged according to the revised RECIST guideline version 1.1 (≥10 mm for lymph nodes) [25].

Third, we propose that a PET-CT should be performed prior to invasive biopsy in cases where the differentiation between benign and malignant lymphadenopathy remains unclear after an ultrasound or CT. Recent clinical studies have reported the significance of a PET-CT for patients with lymphadenopathy after COVID-19 vaccination [26,27]. In our quantitative evaluation using 18F-FDG PET-CT, we first focused on the lymph node with the highest SUVmax and measured its minor axis length, ellipticity, and TBR, even in malignant tumors (except breast cancers). Our subsequent analysis revealed ellipticity and TBR as significant differentiating factors for predicting benign or malignant lymph nodes (Table 4a,b) (*p* = 0.045, 0.051). Considering reproducibility and simplicity, our measurement of ellipticity was conducted in the horizontal section with the greatest minor axis length of the node. Although the SUVmax can vary depending on the imaging equipment and conditions, TBR serves as a semi-quantitative index that minimizes these effects [28]. Accordingly, ellipticity and TBR measurements on a PET-CT can serve as useful tools for distinguishing between MHL and VAHL. The present study is the first to highlight the utility of ellipticity and TBR in differentiating lymphadenopathy following vaccination.

As the conclusion from our study, in patients who present with unilateral axillary lymphadenopathy, inquiries should first be made regarding their vaccination history and timing. Ultrasonography should be performed to assess the presence or absence of a fatty hilum within the lymph nodes. Unilateral lymphadenopathy with fatty hilum can be considered to indicate VAHL. In cases where no fatty hilum is detected, non-contrast CT should be performed to measure the minor axis of the lymph node. As the next step, if MHL should be suspected in cases, a PET-CT scan should be conducted in addition to a non-contrast CT scan to measure ellipticity and the TBR of the lymph node with the highest SUVmax, and a biopsy should be considered for accurate diagnosis.

The present study has several limitations. First, this was a retrospective study with a small sample size, thus weakening the strength of our results. Further prospective studies with a larger sample size are necessary to confirm our findings. Second, in the majority of patients in the VAHL group, the diagnosis was obtained after clinical follow-up and was not confirmed pathologically. It is possible that other benign diseases could have been present after the COVID-19 vaccination.

## Figures and Tables

**Table 1:** Patient characteristics of 3 groups with ultrasonography and/or CT scan.

		ALL (*n* = 188)	VAHL (*n* = 27)	MHL (*n* = 21)	EqHL (*n* = 140)	Pv ^a^
Male/Female		87/101	13/14	2/19	72/68	<0.01
Age Mean (SD), y		63.9 (14.6)	66.6 (14.4)	65.9 (12.8)	63 (14.8)	0.42
Disease, No. (%)	Malignancy					
	Breast cancer	37 (19.7)	3 (11)	17 (81)	18 (12.9)	<0.01
	Lymphoma	27 (14.4)	3 (11)	2 (10)	22 (15.7)	0.66
	Lung cancer	25 (13.3)	7 (26)	1 (5)	17 (12.1)	0.07
	Other malignancy	77 (41)	12 (44)	1 (5)	65 (46.4)	<0.01
	Non-malignancy	23 (12.2)	2 (7)	0 (0)	19 (13.6)	0.15
Timing of imaging test, No. (%)						
	Pre-treatment	113 (60.1)	15 (56)	13 (62)	85 (60.7)	0.87
	During treatment	29 (15.4)	3 (11)	8 (38)	18 (12.9)	<0.01
	Post-treatment	46 (24.4)	9 (33)	0 (0)	37 (26.4)	<0.05
Vaccination, No. (%)		183 (97.3)	27 (100)	16 (76)	140 (100)	<0.01

Categorical variables are reported as frequencies and percentages. Continuous variables are reported as median and interquartile range (IQR). ^a^ *p*-value of the test among the three groups.

**Table 2:** Frequency and chi-square test on fatty hilum presence of 3 groups with ultrasonography and/or CT scan.

	Fatty Hilum, No. (%)		
ALL (*n* = 188)	44	(23.4)	
VAHL (*n* = 27)	8	(30)	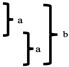
MHL (*n* = 21)	0	(0)	
EqHL (*n* = 140)	36	(25.7)	

^a^ *p* < 0.01, ^b^ Not statistically significant.

**Table 3:** CT imaging assessment: univariate and multivariate logistic regression analyses between VAHL and MHL groups (**a**), and MHL and EqHL groups (**b**).

(**a**)				
	**Univariate**		**Multivariate**	
**Variable**	**Pv**	**OR** **(95% CI)**	**Pv**	**OR** **(95% CI)**
Age, y	0.93	1.00 (0.96–1.04)	0.77	0.99 (0.95–1.04)
Minor axis, mm	<0.01	0.71 (0.51–0.90)	<0.01	0.72 (0.51–0.92)
Ellipticity	0.18	0.14 (0.006–2.43)	0.73	0.56 (0.019–16.23)
(**b**)				
	**Univariate**		**Multivariate**	
**Variable**	**Pv**	**OR** **(95% CI)**	**Pv**	**OR** **(95% CI)**
Age, y	0.59	1.01 (0.98–1.05)	0.79	1.01 (0.97–1.04)
Minor axis, mm	<0.01	1.18 (1.05–1.35)	0.048	1.14 (1.00–1.32)
Ellipticity	0.037	12.4 (1.16–154.99)	0.23	5.1 (0.36–76.71)

**Table 4:** ^18^F-PET-CT imaging assessment: univariate and multivariate logistic regression analyses between VAHL and MHL groups (**a**), and MHL and EqHL groups (**b**).

(**a**)				
	**Univariate**		**Multivariate**	
**Variable**	**Pv**	**OR** **(95% CI)**	**Pv**	**OR** **(95% CI)**
Age, y	0.91	1.00 (0.96–1.05)		
TBR	<0.01	0.78 (0.61–0.95)	0.051	0.81 (0.62–1.00)
Minor axis, mm	<0.01	0.67 (0.48–0.86)	0.082	0.79 (0.55–1.03)
Ellipticity	<0.01	0.002 (<0.001–0.091)	0.045	0.009 (<0.001–0.896)
Blood glucose level, mg/dL	0.93	1.00 (0.96–1.04)		
(**b**)				
	**Univariate**		**Multivariate**	
**Variable**	**Pv**	**OR** **(95% CI)**	**Pv**	**OR** **(95% CI)**
Age, y	0.42	1.01 (0.98–1.05)		
TBR	<0.01	1.18 (1.06–1.33)	0.024	1.17 (1.02–1.34)
Minor axis, mm	<0.01	1.21 (1.06–1.39)	0.63	1.04 (0.88–1.23)
Ellipticity	<0.01	61.39 (4.11–>999)	1.015	45.38 (2.08–>999)
Blood glucose level, mg/dL	0.56	0.99 (0.96–1.02)		

## Data Availability

The datasets analyzed in this study are available from the corresponding author upon reasonable request.
